# Dosimetry after peptide receptor radionuclide therapy: impact of reduced number of post-treatment studies on absorbed dose calculation and on patient management

**DOI:** 10.1186/s40658-020-0273-8

**Published:** 2020-01-23

**Authors:** Alexandre Chicheportiche, Simona Ben-Haim, Simona Grozinsky-Glasberg, Kira Oleinikov, Amichay Meirovitz, David J. Gross, Jeremy Godefroy

**Affiliations:** 1 Department of Nuclear Medicine & Biophysics, Hadassah-Hebrew University Medical Center, 91120 Jerusalem, Israel; 2 Institute of Nuclear Medicine, University College London Hospitals, London, UK; 3 Neuroendocrine Tumor Unit, ENETS Center of Excellence, Endocrinology and Metabolism Department, Hadassah-Hebrew University Medical Center, 91120 Jerusalem, Israel; 4 Oncology Department and Radiation Therapy Unit, Hadassah-Hebrew University Medical Center, 91120 Jerusalem, Israel

**Keywords:** Peptide receptor radionuclide therapy (PRRT), [^177^Lu]-DOTA-TATE, Dosimetry, SPECT/CT

## Abstract

**Background:**

After each cycle of [^177^Lu]-DOTA-TATE peptide receptor radionuclide therapy (PRRT) dosimetry is performed to enable precise calculation of the radiation-absorbed dose to tumors and normal organs. Absorbed doses are routinely calculated from three quantitative single-photon emission computed tomography (SPECT) studies corrected by computed tomography (CT) acquired at *t*_1_ = 24 h, *t*_2_ = 96 h, and *t*_3_ = 168 h after the first cycle of treatment. After following cycles, a single SPECT/CT study is performed. The aim of the present study is to assess the feasibility of a “two time point” quantitative SPECT/CT protocol after the first PRRT cycle and its impact on patient management.

Quantitative SPECT/CT data of 25 consecutive patients with metastatic neuroendocrine tumors after PRRT were retrospectively analyzed. Radiation-absorbed doses calculated using the standard protocol with three SPECT/CT studies acquired at (*t*_1_, *t*_2_, *t*_3_) were compared to those obtained from three different “two time point” protocols with SPECT/CT studies performed at (*t*_1_, *t*_2_), (*t*_1_, *t*_3_), or (*t*_2_, *t*_3_).

**Results:**

The best agreement for the cumulative doses absorbed by the kidneys, bone marrow, liver, spleen, and tumors with the conventional protocol was obtained with the (*t*_1_, *t*_3_) protocol with mean relative differences of − 1.0% ± 2.4%, 0.4% ± 3.1%, − 0.9% ± 4.0%, − 0.8% ± 1.1%, and − 0.5% ± 2.0%, respectively, and correlation coefficients of *r* = 0.99 for all.

In all patients, there was no difference in the management decision of whether or not to stop PRRT because of unsafe absorbed dose to risk organs using either the standard protocol or the (*t*_1_, *t*_3_) protocol.

**Conclusion:**

These preliminary results demonstrate that dosimetry calculations using two quantitative SPECT/CT studies acquired at 24 and 168 h after the first PRRT cycle are feasible and are in good agreement with the standard imaging protocol with no change in patient management decisions, while enabling improved patient comfort and reduced scanner and staff time.

## Background

^177^Lu-DOTATATE has been proven to be an effective therapy of neuroendocrine tumors [1–3]. Commonly, recommended schedule of treatment with [^177^Lu]-DOTA-TATE consists of four fixed cycles of 7.4 GBq (200 mCi) infusions every 6–12 weeks [1, 4–6]. This so-called empiric protocol is in accordance with the Food and Drugs Administration approval and the European Medicines Agency summary of product characteristics [7, 8]. The amount of ^177^Lu radioactivity administered has to achieve an optimal therapeutic effect of radioligand therapy, leading to a maximal absorbed dose in the tumors with limited side effects to radiosensitive organs, namely the kidneys and bone marrow. The true threshold of the kidney-absorbed dose that predisposes patients to toxicity is unknown. Based on data of external beam radiation and PRRT with ^90^Y-labeled analogs, commonly accepted values vary from 18 to 30 Gy [9–15]. However, there is growing evidence that PRRT with [^177^Lu]-octreotate is less nephrotoxic [16, 17]. In a study of 323 patients, where a median number of four 7.4 GBq cycles have been administered, Bergsma et al. [16] did not observe subacute grade 3 or 4 nephrotoxicity. Moreover, no correlation was observed between radiation-absorbed dose to the kidneys and worsening creatinine clearance on long-term follow-up. For bone marrow, the commonly accepted threshold is 2 Gy (based on historical cohorts of patients treated with ^131^I [18]), and here too the clinical relevance in PRRT with ^177^Lu-Octreotate was not proven. Bergsma et al. [19] did not find any correlation between cumulative bone marrow-absorbed dose and therapy-related persistent hematologic disorder. Moreover, a wide heterogeneity between authors in the methodology of bone marrow dosimetry (planar, SPECT, blood samples) makes the comparison between studies difficult. In practice, it has been pointed out by Sandström [11] that the absorbed dose to bone marrow is rarely a limiting factor (1.5% of the patients) when using a 23 Gy safety threshold for kidneys.

Nevertheless, in keeping with the current European Council Directive 2013/59 (Article 56) [20, 21] and EANM/MIRD guidelines for Quantitative ^177^Lu SPECT [22], dosimetry is performed in our institution. Treatment is stopped if the cumulative absorbed dose to kidneys is expected to exceed 25 Gy and the cumulative bone marrow-absorbed dose is expected to exceed 2 Gy, unless otherwise decided by a multidisciplinary team based on assessment of the individual benefit/risk ratio. Therefore, individual dosimetry is performed after each PRRT cycle to evaluate the cumulative absorbed dose to the kidneys and bone marrow, to estimate the tumor-absorbed dose, and to decide whether further therapy cycles can be administered safely.

In order to estimate the pharmacokinetics of [^177^Lu]-DOTA-TATE and to calculate the radiation doses absorbed by the patient’s organs and tumors, quantitative single-photon emission computed tomography (SPECT) images corrected for photon attenuation (from CT attenuation maps), scatter photons, and blurring are acquired [22–24]. Following the EANM/MIRD guidelines [22], three SPECT/CT studies, at *t*_1_ = 24 h, *t*_2_ = 96 h, and *t*_3_ = 168 h are acquired after the first cycle of treatment in order to model the source organ/tumor distribution. For the following cycles, only a single SPECT/CT study at *t* = 24 h is necessary assuming an unchanged effective half-life of [^177^Lu]-DOTA-TATE [25, 26], a good example of tradeoff between feasibility and desirable accuracy.

With the intention to avoid patients returning to the hospital several times following the [^177^Lu]-DOTA-TATE injection for several post-treatment scans, different methods have been suggested in the literature. A recent study by Willowson et al. [26] investigated the feasibility of reliable renal dosimetry using a single time point imaging from the second cycle of treatment, as proposed before by Garske et al. [25], or even from the first treatment cycle assuming an average patient effective half-life. Hänscheid et al. [27] showed that the use of a single SPECT/CT acquired 4 days after injection was sufficient to derive reliable renal-absorbed dose estimates. Sundlöv et al. [28] considered different alternative treatment planning strategies to hybrid planar-SPECT/CT method, each representing a simplification in terms of image acquisition and dosimetric calculations. Heikkonen et al. [29] previously demonstrated the feasibility to estimate the kidney dosimetry using two SPECT/CT studies acquired after *t*_1_ = 24 h and *t*_3_ = 168 h. However, they recommend acquiring two SPECT/CT studies after each cycle of treatment.

In present study, we have assessed the feasibility of three different “two time point” protocols, where two quantitative SPECT/CT studies are acquired at (*t*_1_, *t*_2_), (*t*_1_, *t*_3_), or (*t*_2_, *t*_3_) after the first cycle of treatment, and a single study is performed after each following therapy. We focused on the influence of the “two time point” protocols on the dose absorbed by the kidneys as well as changes in the tumor, bone marrow, liver, and spleen dosimetry.

## Methods

### Patients

Between November 7, 2017, and May 21, 2019, 111 PRRT treatment cycles with [^177^Lu]-DOTA-TATE were administered to 45 consecutive patients at our institution. Inclusion criteria for this study were as follows: (a) age ≥ 20 years, (b) patients who started and completed their series of treatments during this time period, and (c) patients for whom the sole reason of treatment discontinuation was treatment toxicity (hematotoxicity, although not reflected by the bone marrow dosimetry or general deterioration) or an expected absorbed dose > 25 Gy to kidneys and > 2 Gy to bone marrow.

**Fig. 1:**
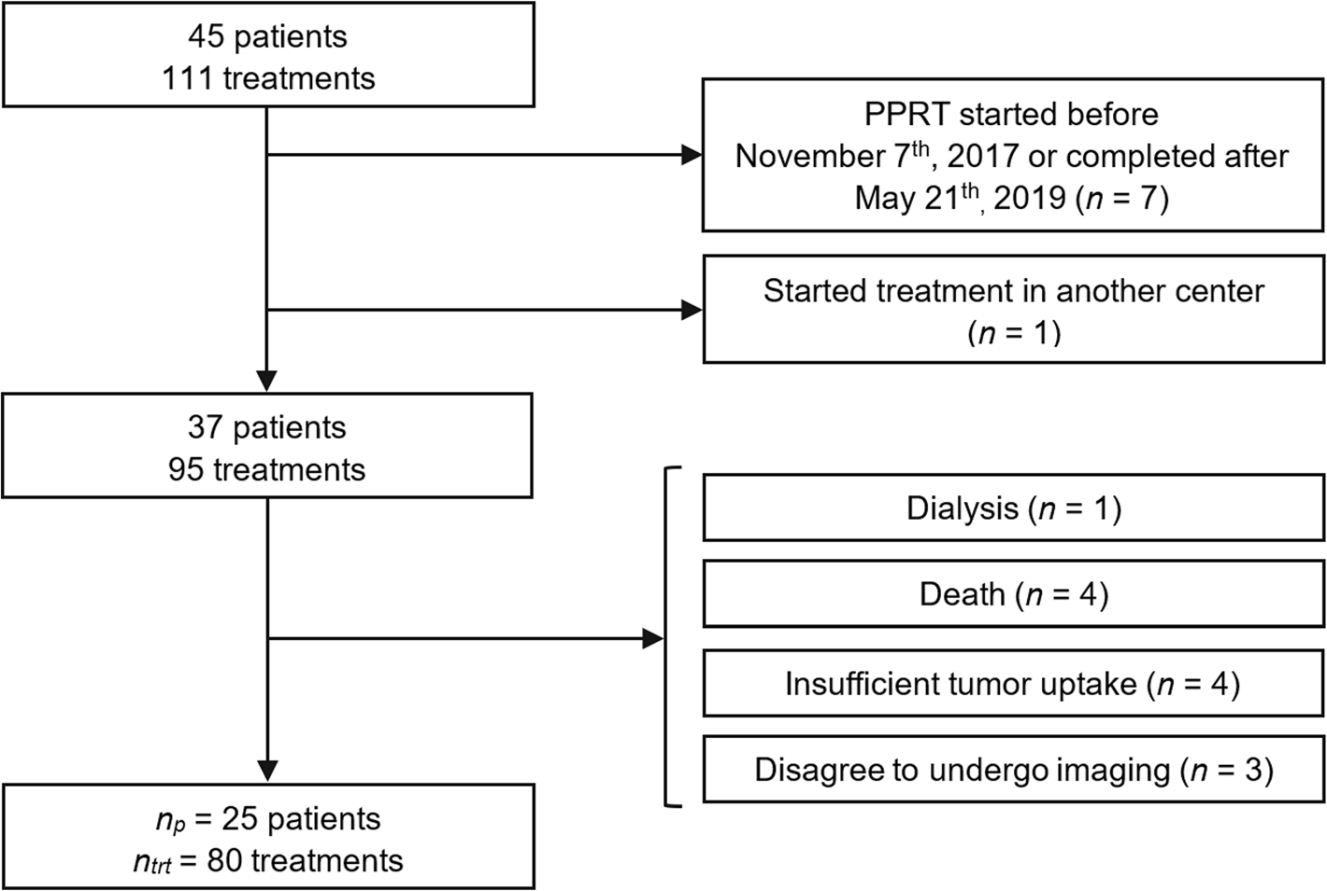
Chart of patient inclusion. *n*_*p*_ represents the number of patients included in the study and *n*_*trt*_ is the corresponding total number of therapy cycles

Thirty-seven of these 45 patients started and completed their therapy during this period. Four patients were excluded because of an insufficient uptake of the radiopharmaceutical in tumors, and four others died before completing the series of treatment. Three patients who refused to undergo the series of post-treatment scans and received a single cycle of treatment were also excluded from the study. One additional patient with chronic renal failure under dialysis was excluded. The remaining 25 patients (14 men, 11 women; average age 61 years, range 34–86 years) were included in this single-center retrospective study (Fig. 1). A total of 80 therapy cycles were administered (12 patients received 4 cycles, 9 received 3 cycles, 1 received 2 cycles, and 3 received a single therapy cycle). Patient clinical characteristics are summarized in Table 1.

**Table 1:** Clinical characteristics

Characteristic	Value
Total number of patients	25
Age (years)	
Mean± standard deviation	61± 12
Range	34–86
Gender	
Male	14
Female	11
Primary tumor site	
Pancreas	8
Stomach	1
Small bowel	6
Neck	1
Thymus	1
Rectum	1
Carotid glomus	1
Adrenal glands	1
Retroperitoneum	1
Lung	2
Unknown	2
Sites of metastases	
Liver	18
Lymph nodes	15
Bone	11
Lung	1
Peritoneum	4
Pancreatic bed	1

At our institution, before the administration of any subsequent treatment cycle, the absorbed doses to kidneys during the previous (*p*) treatments are considered to calculate the “expected” cumulative absorbed dose to kidneys after the following (*p* + 1) cycle. The latter is calculated as the mean absorbed dose over the previous (*p*) treatments to which is added the cumulative absorbed dose over these (*p*) treatments. When the cumulative absorbed kidney dose after the subsequent cycle was expected to exceed 25 Gy, no further PRRT was given, unless decided otherwise by a multi-disciplinary team. Of the 13 patients who did not complete 4 cycles of PRRT, in 8/13 (62%), therapy was stopped because the kidney-absorbed dose after the following cycle was expected to exceed 25 Gy. In the remaining 5/13 (38%) patients, therapy was stopped because of general deterioration.

Recently, we published a more precise management approach for empirical treatments based on the “predicted” kidney-absorbed dose after the first(s) treatment cycles allowing for an early decision regarding the number of cycles that may be given [30]. For instance, using a safety threshold of 25 Gy, patients whose absorbed dose to kidneys after the first cycle of treatment D_1_ is below 5.6 Gy can receive safely four cycles of treatment. For patients with *D*_1_ > 5.6 Gy, the incremental amount of information stemming from the absorbed dose D_2_ after the second cycle may alleviate the need for further follow-up of kidney dosimetry. This management method is used routinely from March 2019 in our department. Although all the patients included in this study started their treatment before this date and underwent dosimetry over all their therapy cycles, the “predicted” absorbed dose management approach has also been applied retrospectively to investigate the impact on patient management when reducing time points.

### PRRT therapy

DOTA-(Tyr3)-Octreotate GMP was purchased either from ABX (Radeberg, Germany) or Auspep (Tullamarine, Australia). ITG (Munich, Germany) supplied non-carrier-added ^177^LuCl_3_.

[^177^Lu]-DOTA-Octreotate was locally prepared by S.R.Y Ltd. (Jerusalem, Israel). All batches passed high-performance liquid chromatography quality control.

Infusion of 1.5 L of amino acids solution (Vamin 18 g N/L electrolyte-free, Fresenius Kabi) started at least half an hour prior to administration of the radiopharmaceutical and continued for several hours (4–6 h). The radioactive ligand, diluted in 200 ml of saline, was co-administered intravenously over a period of 30 min. The mean activity per cycle of treatment was 7.3 ± 0.32 GBq (198.1 ±8.8 mCi) with a median cumulative activity per patient of 23.0 GBq (7.1–30.0 GBq). The interval between treatment cycles was 6–12 weeks (median = 8 weeks).

### Post-treatment imaging

Post-treatment studies (PTS) acquired after each cycle of treatment include a planar whole-body study and a quantitative SPECT/CT study of the abdomen covering at least the kidneys, liver, and spleen. An additional field of view (FOV) of the region including the tumor(s) was acquired if not within the liver/kidney FOV. Serial SPECT/CT studies were acquired at *t*_1_ = 20 ± 2 h, *t*_2_ = 97 ± 11 h, and *t*_3_ = 160 ± 7 h after injection of the first therapeutic dose. After subsequent cycles, a single SPECT/CT was acquired at 20 ± 4 h after therapy administration.

Studies were acquired on a Discovery NM/CT 670 scanner (International General Electric, General Electric Medical Systems, Haifa, Israel). This system combines a dual-head coincidence SPECT camera with an axial FOV of 40 × 54 cm, a NaI(Tl) crystal thickness of 9.5 mm, and 59 photomultiplier tubes. All images were acquired with a 20% energy window around the main photopeak of ^177^Lu (208 keV; 10.4% probability) [31] with Medium Energy General Purpose collimators. For scatter estimation, the dual energy window method was used using an energy window placed ± 10% around 166.4 keV. Whole body images were acquired with step-and-shoot mode (180 s per view) in a 256 × 1024 matrix, with zoom 1.0 and body contour. SPECT imaging was performed applying 60 views over 360° (30 angular steps per head, 6° angle step) with a 30-s exposure per frame (15 min acquisition/FOV) in a 128 × 128 matrix size (4.4 mm pixels), with zoom 1.0 and body contour. CT was acquired before each SPECT acquisition with the integrated BrightSpeed multidetector CT (24 rows—maximum 16 slices/rotation) using a tube voltage of 120 kV and the Smart current option (80–220 mA—noise index, 17).

SPECT images were calibrated as previously described [30]. Briefly, calibration of SPECT images was based on a series of SPECT acquisitions of a 20-mL vial placed in the center of the gamma camera FOV with a ^177^Lu activity ranging from 114.7 MBq (3.1 mCi) to 7215 MBq (195 mCi). The ^177^Lu calibration source was placed in the center of 8 1-L saline bags with two additional ^177^Lu sources in order to simulate an amount of scatter similar to a clinical scan.

### Image analysis and dosimetry calculation

Image analysis for dosimetry was performed using the General Electric Dosimetry Toolkit (DTK) software [32] available for the Xeleris 3.0 Workstation (International General Electric, General Electric Medical Systems, Haifa, Israel) where images are reconstructed with the ordered-subsets expectation maximization algorithm (2 iterations, 10 subsets), attenuation correction (from CT attenuation maps), scatter correction, and resolution recovery (for blurring).

Processing with GE DTK consists of delineation of the organs and tumors on functional (SPECT) or anatomical (CT) images. Volumes of interest (VOIs) were placed over the whole healthy organs of interest (kidneys, liver, spleen, and remainder of the body) and over tumors. Reference [33] provides more precision about image processing using GE DTK.

Radiation-absorbed doses were computed using an in-house interactive data language (IDL) code developed in our department. The code takes as input data the output file of the GE DTK software including the volume and the activity concentrations in each drawn VOIs and at each time point. The code then performs mono-exponential curves fitting (from multiple time points after the first cycle and from a single time point for the following cycles, assuming no changes in the effective half-life for organs and tumors of interest [25, 26]), numerical integrations, and dosimetry calculation. Radiation-absorbed doses by the tumors were computed using the method proposed by Sandström et al. [23] where self-doses only are taken into account. The tumor-absorbed doses (mGy) were obtained by the multiplication of the residence time of the radioactivity concentration in the tumor ([MBq · s]/[MBq · kg]) by an appropriate dose conversion factor DCF_tumor_ = 0.0236 [mGy · g]/[MBq · s] (renamed later ACDF (activity concentration dose factor) by Sandström et al. in [34]) and by the administered activity A_adm_ (MBq). For healthy organs (kidneys, liver, spleen, bone marrow, remainder of the body), the medical internal radiation dose (MIRD) formalism [35] was used with dose factors taken from OLINDA/EXM 1.0 [36] for the adult male and adult female phantoms. More details are given in reference [33].

In addition to the standard protocol using three time points (*t*_1_, *t*_2_, *t*_3_) for the fitting of the time activity curves, the dosimetry calculation was also performed with different “two time point” protocols with SPECT/CT studies performed at (*t*_1_, *t*_2_), (*t*_1_, *t*_3_), or (*t*_2_, *t*_3_). For all of these protocols, the output file of DTK obtained with the standard protocol has been used and the IDL code has been modified in order to take into account two time points only for mono-exponential curves fitting. Therefore, the differences in dosimetry results between the different methods presented are only due to the different time points used with no differences in processing.

### Bone marrow dosimetry

Bone marrow dosimetry was calculated as previously described [33]. Briefly, in order to quantify the self-dose to bone marrow, blood samples were drawn after the first (24 h) and third (168 h) SPECT/CT studies performed after the first injection of the radiopharmaceutical. No additional samples have been drawn for all the 25 patients due to organizational constraints in our department. The blood activity concentration has been fitted by a mono-exponential curve and integrated to infinity in order to estimate the residence time and then the self-dose to the bone marrow, assuming that the activity concentration in the latter is the same as in the blood [37]. Therefore, in the scope of this study, the influence of the “two time point” protocols on bone marrow dosimetry will reflect only the cross-dose contribution.

For the following cycles, a blood sample was drawn approximately 20 h after the administration of [^177^Lu]-DOTA-TATE.

### Statistical methods

Differences between the results obtained using the standard “three time point” protocol and the “two time point” protocols were assessed with Bland and Altman plots. The correlation between the different methods was assessed with Pearson’s correlation coefficient *r* and the angular coefficient *a* (slope of the linear regression line).

To test the power of the null hypothesis, i.e., that there is no difference in the management of patients whether decisions were made using the standard protocol or one of the “two time point” protocols, a one-sided binomial test were performed using the StatXact, Cytel Inc., Cambridge MA, version 10 20 software.

## Results

**Fig. 2:**
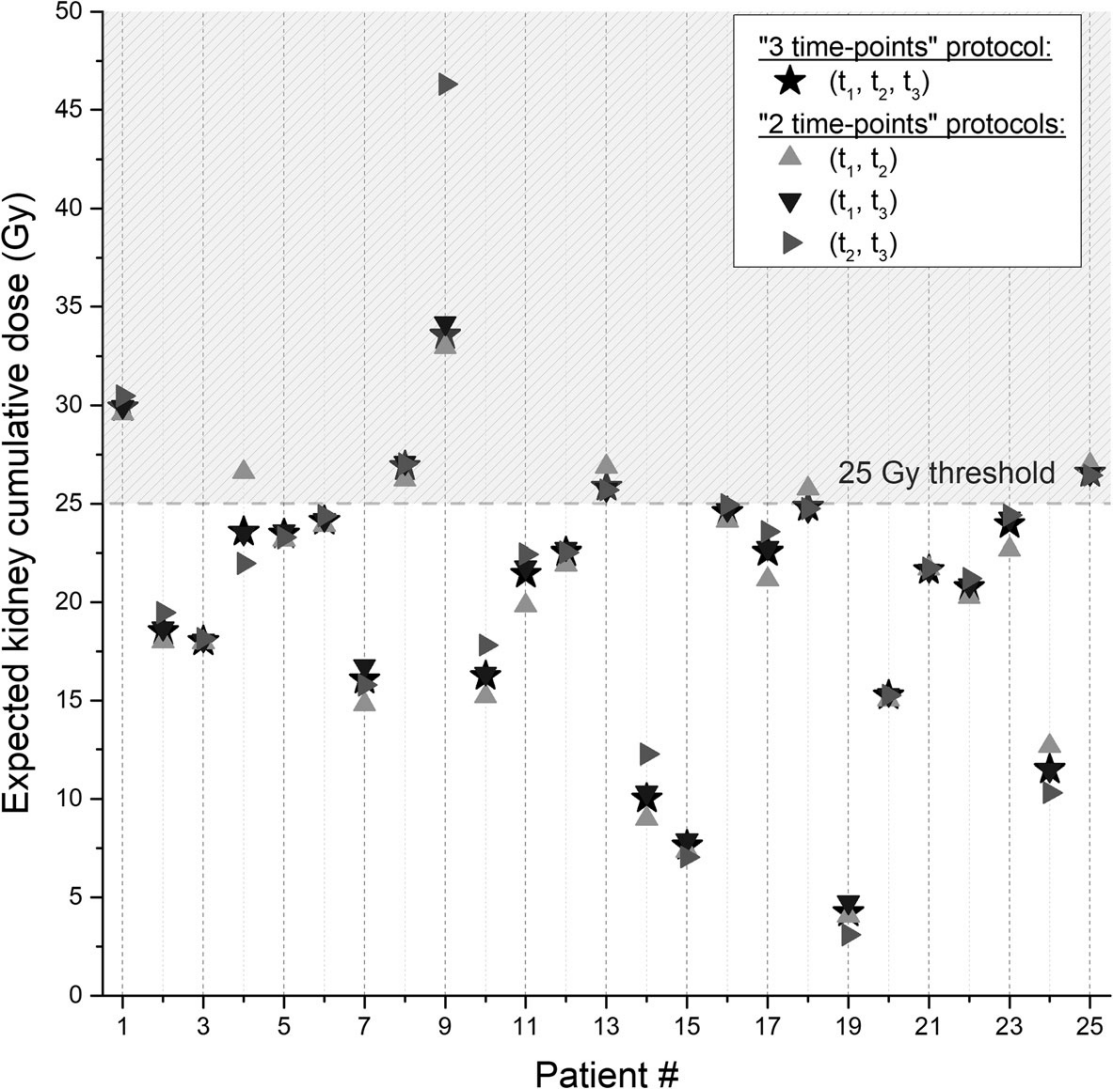
Expected dose after the last PRRT calculated from the previous ones (1 to 3) using the standard “3 time-points” protocol (black stars) or the different “2 time-points’ protocols (triangles). Each vertical line represents a patient and the dashed region the unsafe dose region and interruption of the treatment (patients # 1, 8, 9, 13, and 25 following the standard protocol)

Figure 2 shows the “expected” kidney cumulative absorbed doses calculated using the standard “three time point” protocol and the different “2 time-points” protocols. There was no change in patient management based on the (*t*_1_, *t*_3_) or (*t*_2_, *t*_3_) protocols compared to the standard management based on the SPECT/CT studies acquired at (*t*_1_, *t*_2_, *t*_3_). Indeed, all the patients who continued or discontinued therapy according to the standard protocol would have been managed similarly with the (*t*_1_, *t*_3_) or (*t*_2_, *t*_3_) protocol. However, in 2/25 cases, the patient management using the (*t*_1_, *t*_2_) protocol would have been different from the standard one. The (*t*_1_, *t*_2_) protocol led also to a wrong patient management of one patient by overestimating the bone marrow expected cumulative absorbed dose.

**Fig. 3:**
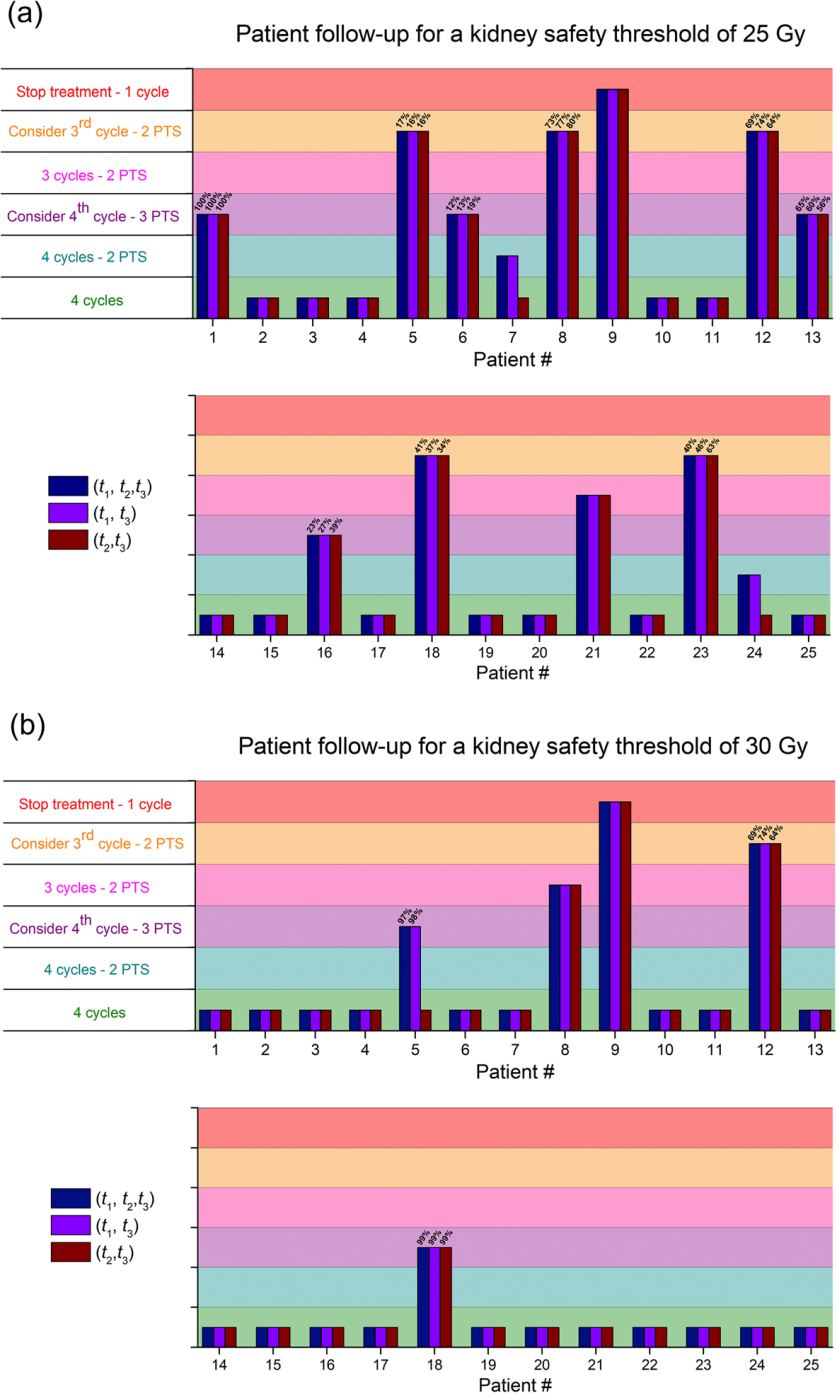
Management of the 25 patients included in the study based on the kidney cumulative dose “predicted” after one, two, or three post-treatment scan(s) (PTS) and calculated either by using the standard protocol or by using the (*t*_1_, *t*_3_) or (*t*_2_, *t*_3_) “2 time-points” protocols with a safety threshold fixed at **a** 25 Gy and **b** 30 Gy. Each color bar represents different protocol. Horizontal lines delimit the different managements of the patient (4 CYCLES: the patient can receive 4 cycles of treatment safely, 1 PTS only; 4 CYCLE—2 PTS: the patient can receive 4 cycles of treatment safely, 2 PTS only; CONSIDER A fourth CYCLE—3 PTS: consider to administer a fourth cycle of treatment based on the probability to exceed the safety threshold after 4 cycles; 3 CYCLES—2 PTS: the patient can receive only 3 cycles of treatment safely, 2 PTS only; CONSIDER A third CYCLE—2 PTS: consider to administer at maximum a third cycle of treatment based on the probability to exceed the safety threshold after 3 cycles; STOP TREATMENT—1 cycle: stop treatment after a single cycle of treatment)

In a previous paper [30], we proposed a simplified algorithm for the follow-up of kidney-absorbed dose, taking advantage of the predictive power of the post treatment scan after the first or two first courses of [^177^Lu]-DOTA-TATE. This algorithm was used here with the standard and with the (*t*_1_, *t*_3_) or (*t*_2_, *t*_3_) protocols, using a 25 Gy or a 30 Gy safety threshold (Fig. 3a, b). Patient management based on the (*t*_1_, *t*_3_) protocol would have been similar to their management based on the standard protocol. However, using the (*t*_2_, *t*_3_) protocol, management would have changed. For instance, for patient #5, by using a 30 Gy threshold, the standard or the (*t*_1_, *t*_3_) protocols predict, after three cycles and three post-treatment scans, a risk of 97% and 98% respectively to exceed this threshold after a fourth cycle, whereas according to the (*t*_2_, *t*_3_) protocol, four therapy cycle could be safely administered.

**Fig. 4:**
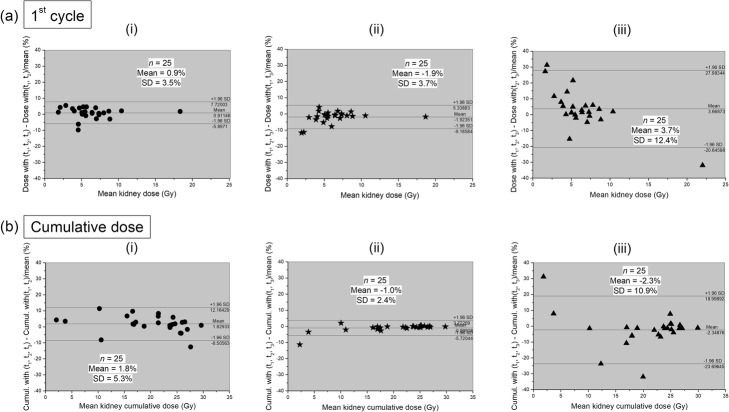
Bland and Altman plots showing differences in the kidney-absorbed dose **a** after the first PRRT cycle and **b** after completion of the treatment, computed using the standard protocol and the “2 time-points” (i) (*t*_1_, *t*_2_), (ii) (*t*_1_, *t*_3_), or (iii) (*t*_2_, *t*_3_) protocols, with 95% limits of agreement (mean ± 1.96 SD)

**Fig. 5:**
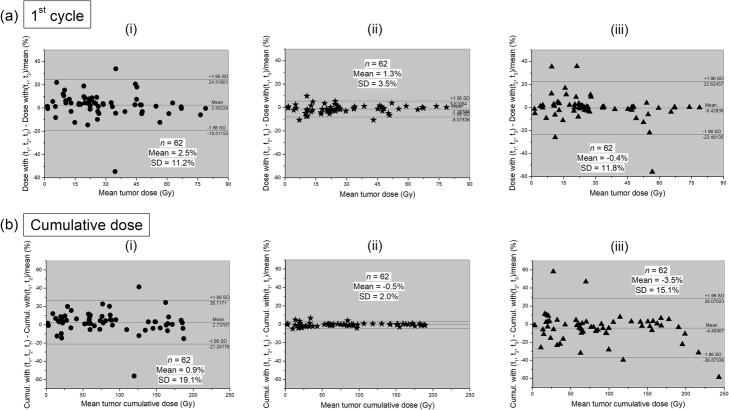
Bland and Altman plots showing differences in the dose absorbed by tumors **a** after the first PRRT cycle and **b** after completion of the treatment, computed using the standard protocol and the “2 time-points” (i) (*t*_1_, *t*_2_), (ii) (*t*_1_, *t*_3_), or (iii) (*t*_2_, *t*_3_) protocols, with 95% limits of agreement (mean ± 1.96 SD). For clarity reason, an outlier cumulative tumor-absorbed dose point calculated with the (*t*_1_, *t*_2_) protocol is not shown at (*x*, *y*) = (81 Gy, − 113%).

Differences between the kidney-absorbed dose (a) after the first cycle of therapy and (b) after completion of the treatment using the “2 time-points” (i) (*t*_1_, *t*_2_), (ii) (*t*_1_, *t*_3_), or (iii) (*t*_2_, *t*_3_) protocols and the standard (*t*_1_, *t*_2_, *t*_3_) protocol are shown in Fig. 4. Differences in tumor-absorbed doses are shown in Fig. 5. As shown above, only the (*t*_1_, *t*_3_) protocol led to similar management decisions than those based on the standard acquisition protocol. It is noteworthy that there is excellent agreement between the (*t*_1_, *t*_3_) protocol and the standard one with a mean relative difference of − 1.9% ± 3.7% for the kidney-absorbed dose after the first cycle and − 1.0% ± 2.4% for the 25 cumulative kidney-absorbed doses and with a Pearson’s correlation coefficient *r* = 0.99 (all *P* < 0.0001). Similarly for tumors, the mean differences for 62 lesions were − 1.3% ± 3.5% after the first therapy cycle and − 0.5% ± 2.0% for the cumulative absorbed doses, all with *r* = 0.99 (*P* < 0.0001). The mean differences in the cumulative absorbed dose to the bone marrow, liver, and spleen calculated with (*t*_1_, *t*_3_) compared to the standard protocol were − 0.4% ± 3.1%, − 0.9% ± 4.0%, and − 0.8% ± 1.1% respectively, all with *r* = 0.99 (*P* < 0.0001).

Cumulative absorbed doses calculated with the two other “2 time-points” protocols were generally higher. For example, the cumulative absorbed doses to kidneys calculated with the (*t*_1_, *t*_2_) or (*t*_2_, *t*_3_) protocols were similar with a mean relative error of 1.8% ± 5.3% and − 2.3% ± 10.9% respectively from the absorbed doses calculated in the standard way. The mean relative errors in cumulative absorbed doses, Pearson’s coefficients *r*, and angular coefficients *a* obtained for the kidneys, bone marrow, liver, spleen, and tumors are summarized in Table 2. Note that the “2 time-points” (*t*_1_, *t*_3_) protocol presents the smallest errors from the standard EANM/MIRD acquisition dosimetry protocol [22].

**Table 2:** Angular coefficients *a*, Pearson’s coefficients *r*, and mean relative errors in cumulative doses from the standard calculation obtained for the kidneys, bone marrow, liver, spleen, and tumors when calculated using the three different “2 time-points protocols”

Cumulative doses calculated with			
	(*t*_1_, *t*_2_)	(*t*_1_, *t*_3_)	(*t*_2_, *t*_3_)
Kidneys	1.8% ± 5.3%	− 1.0% ± 2.4%	− 2.3% ± 10.9%
	*r* = 0.98	*r* = 0.99	*r* = 0.97
	*a* = 1.02	*a* = 1.00	*a* = 0.97
Bone marrow	− 0.8% ± 16.7%	0.4% ± 3.1%	− 1.7% ± 13.4%
	*r* = 0.95	*r* = 0.99	*r* = 0.96
	*a* = 1.35	*a* = 1.00	*a* = 0.90
Liver	3.4% ± 22.8%	− 0.9% ± 4.0%	− 18.5% ± 24.4%
	*r* = 0.97	*r* = 0.99	*r* = 0.93
	*a* = 1.57	*a* = 1.00	*a* = 0.77
Spleen	6.8% ± 6.9%	− 0.8% ± 1.1%	− 8.1% ± 9.8%
	*r* = 0.99	*r* = 0.99	*r* = 0.98
	*a* = 0.92	*a* = 1.00	*a* = 1.09
Tumors	0.9% ± 19.1%	− 0.5% ± 2.0%	− 3.5% ± 15.1%
	*r* = 0.96	*r* = 0.99	*r* = 0.90
	*a* = 0.97	*a* = 1.00	*a* = 1.19

Analyzing the measured effective half-lives for healthy organs and tumors, the same observations have been done. Using the standard protocol half-lives of 53.6 ± 15.8 h, 72.8 h ± 18.4 h, 75.5 h ± 19.1 h, and 92.2 h ± 39.6 h have been respectively obtained for the kidneys, liver, spleen, and tumors. Calculation of these half-lives using the (*t*_1_, *t*_2_), (*t*_1_, *t*_3_), or (*t*_2_, *t*_3_) protocols respectively led to mean relative errors from the standard protocol half-lives of 3.7% ± 10.7%, − 0.1% ± 0.4%, and − 6.1% ± 14.2% for the kidneys, 7.3% ± 30.7%, − 0.9% ± 2.6%, and − 18.0% ± 30.0% for the liver, 9.5% ± 10.0%, − 0.1% ± 0.5%, and − 12.8% ± 14.3% for the spleen, and 7.7% ± 19.0%, − 0.1% ± 0.7%, and − 11.9% ± 23.8% for tumors.

## Discussion

Our null hypothesis was there is no difference in the management of patients whether decisions were made using the standard protocol or at least one of the “2 time-points” protocols. Of the three different “2 time-points” protocols, only management decisions based on the (*t*_1_, *t*_3_) protocol were similar to those based on the standard acquisition protocol. A sample size of 25 patients has a power of 92.8% to detect a true difference of at least 10% at the 0.05 level of significance, using a one-sided binomial test.

The results presented in this work demonstrate that the kidney, bone marrow, liver, spleen, and tumor dosimetry using two SPECT/CT studies acquired 24 h and 168 h after the first administration of the radiopharmaceutical, and a single dosimetry study 24 h after subsequent cycles are feasible, showing a good agreement with the standard protocol, with no change in patient management decisions. Therefore, this protocol can be considered for replacement of the current standard acquisition and dosimetry protocol. This can greatly improve the comfort of the patients not having to return to the hospital for imaging as well as reduce scanner and technologist time. Heikkonen et al. [29] have previously demonstrated the feasibility of using two SPECT/CTs after each and every cycle to estimate kidney dosimetry only. Here, we extended the results to others organs, that may become relevant in future, when personalized dosimetry-based treatments with variable activities will be given or when higher safety thresholds to kidneys will be used.

The use of only two points instead of three for kinetics determination necessarily increases the uncertainty of dosimetry calculations. However, the mean relative errors obtained using the “2 time-points” (*t*_1_, *t*_3_) protocol compared to the standard protocol were less than 1%. In view of the intra-observer reproducibility of − 6.50 ± 6.76% for kidney-absorbed dose estimation [33], determined in preset study when the same operator has drawn twice the kidney VOIs, the errors obtained here are negligible and similar to the inter-observer variability of − 0.98 ± 3.40% [33] (kidney VOIs drawn by two different operators). For the two other “2 time-points” (*t*_1_, *t*_2_) and (*t*_2_, *t*_3_) protocols, mean absolute relative errors of 3% and 7% in average were obtained, respectively. Moreover, dosimetry calculations with these two protocols did not lead to the correct patient management decisions and therefore cannot substitute the current protocol. From a mathematical standpoint, it is intuitive and easy to show that the farther two time points are from one another, the closest the mono-exponential fit estimation will be from the pharmacokinetics obtained with three time points.

As mentioned in the method section, for bone marrow dosimetry, only two blood samples were drawn 24 h and 168 h after the first injection of the radiopharmaceutical due to organizational constraints in our department. The results of this work showing a very low deviation between the “3 time-points” and the (*t*_1_, *t*_3_) protocols give us more confidence in the method used.

Alternatives for reducing the number of post-treatment scans have been investigated. Willowson et al. [26] showed that the use of an average kidney effective half-life leads to a minimum mean relative error of 30% in the absorbed dose estimation. This error is much higher than the deviations presented in this paper. Moreover, in view of the variability of the effective half-lives for other organs and tumors, similar or even higher deviations in absorbed dose calculation from patient specific data are expected. Hänscheid et al. [27] showed that the use of a 96-h post-treatment SPECT/CT leads to reliable integral time estimation with median errors of 5% for the kidneys, 6% for the liver, 8% for the spleen, and 6% for the lesions. Relative errors obtained here are generally lower apart when using the (*t*_2_, *t*_3_) protocol for liver-absorbed dose estimation. Finally, Willowson et al. [26] also investigated the use of complete data for cycle 1 (3 PTS) and of a single time point for the following cycles. This method leads to relative errors of 2 ± 16% in the kidney-absorbed dose estimation. Errors in the cumulative kidney-absorbed dose calculation obtained in the present study when using two time points instead of three were generally lower.

There are several limitations to our study. First, only 25 patients were included in this work. This limitation is due to recent organizational changes in our department in order to perform SPECT/CT studies according to the EANM/MIRD recommendations. Assessment in a larger patient cohort would allow a more certain implementation in clinical practice. Second, our study was done in the framework of the empiric protocol where up to 4 cycles of a fixed activity of 7.4 GBq are administered. However, given the low mean relative errors obtained here, the conclusions of this work should not be different when more than 4 cycles of treatment and/or variable doses higher than 7.4 GBq will be administered or when the 25 Gy kidney safety threshold will be reconsidered.

## Conclusion

These preliminary results demonstrate that dosimetry calculations using two quantitative SPECT/CT studies acquired at 24 and 168 h after the first PRRT cycle are feasible and are in good agreement with the standard imaging protocol with no change in patient management decisions, while enabling improved patient comfort and reduced scanner and staff time.

## Data Availability

Patient imaging was done in the scope of the routine clinical diagnostic studies, and the raw data are stored in the hospital archiving system at the Hadassah-Hebrew University Medical Center, Jerusalem, Israel.

## Abbreviations

CT: Computed tomography
FOV: Field of view
IDL: Interactive data language
MIRD: Medical internal radiation dose
PRRT: Peptide receptor radionuclide therapy
PTS: Post-treatment scans
SPECT: Single-photon emission computed tomography
VOI: Volume of interest
